# Ca^2+^-signaling in airway smooth muscle cells is altered in T-bet knock-out mice

**DOI:** 10.1186/1465-9921-7-33

**Published:** 2006-02-23

**Authors:** Albrecht Bergner, Julia Kellner, Anita Kemp da Silva, Fernando Gamarra, Rudolf M Huber

**Affiliations:** 1 Pneumology, Medizinische Klinik-Innenstadt, Ludwig-Maximilians-University, Munich, Germany

## Abstract

**Background:**

Airway smooth muscle cells (ASMC) play a key role in bronchial hyperresponsiveness (BHR). A major component of the signaling cascade leading to ASMC contraction is calcium. So far, agonist-induced Ca^2+^-signaling in asthma has been studied by comparing innate properties of inbred rat or mouse strains, or by using selected mediators known to be involved in asthma. T-bet knock-out (KO) mice show key features of allergic asthma such as a shift towards T_H_2-lymphocytes and display a broad spectrum of asthma-like histological and functional characteristics. In this study, we aimed at investigating whether Ca^2+^-homeostasis of ASMC is altered in T-bet KO-mice as an experimental model of asthma.

**Methods:**

Lung slices of 100 to 200 μm thickness were obtained from T-bet KO- and wild-type mice. Airway contraction in response to acetylcholine (ACH) was measured by video-microscopy and Ca^2+^-signaling in single ASMC of lung slices was assessed using two-photon-microscopy.

**Results:**

Airways from T-bet KO-mice showed increased baseline airway tone (BAT) and BHR compared to wild-type mice. This could be mimicked by incubation of lung slices from wild-type mice with IL-13. The increased BAT was correlated with an increased incidence of spontaneous changes in intracellular Ca^2+^-concentrations, whereas BHR correlated with higher ACH-induced Ca^2+^-transients and an increased proportion of ASMC showing Ca^2+^-oscillations. Emptying intracellular Ca^2+^-stores using caffeine or cyclopiazonic acid induced higher Ca^2+^-elevations in ASMC from T-bet KO- compared to wild-type mice.

**Conclusion:**

Altered Ca^2+^-homeostasis of ASMC contributes to increased BAT and BHR in lung slices from T-bet KO-mice as a murine asthma model. We propose that a higher Ca^2+^-content of the intracellular Ca^2+^-stores is involved in the pathophysiology of these changes.

## Background

Airway smooth muscle cells (ASMC) mediate bronchial narrowing and therefore play a key role in bronchial hyperresponsiveness (BHR). In asthma, increased ASMC mass has been reported by several investigators [1-3]. However, the question arises whether changes in contractile properties of the ASMC [4] or in the signaling cascades that mediate agonist-induced contraction might also contribute to asthma [5]. Calcium is a ubiquitous signaling molecule that is involved in the regulation of a broad variety of cellular events in almost all mammalian cell types [6-9]. In ASMC, contractile stimuli promote contraction by elevating cytoplasmic calcium-concentration ([Ca^2+^]_c_). Calcium binds to calmodulin, thereby activating myosin light chain (MLC) kinase, which phosphorylates MLC and initiates actomyosin cross-bridge cycling. Consequently, agonist-induced Ca^2+^-signaling has been regarded as a possible target in the pathophysiology of BHR [10,11]. Indeed, in hyperresponsive inbred rats enhanced Ca^2+^-mobilization was correlated with BHR [12,13], though, on the other hand, differences in acetylcholine (ACH)-induced airway narrowing were not associated with differences in ACH-induced Ca^2+^-signaling when we compared three different mouse strains [14]. However, several studies have shown that Ca^2+^-responses can be modulated by a multitude of stimuli including the cytokines IL-1β, TNF-α, IL-4 and IL-13 [15-22]. In addition, CD38/cyclic ADP-ribose-mediated Ca^2+^-signaling has been found to contribute to ASMC hyperresponsiveness [23,24]. But, in all of these studies BHR was investigated either by comparing innate properties of inbred strains or by using single mediators known to be involved in asthma.

T-bet is a T_H_1-specific transcription factor, which has the ability to direct T_H_2- into T_H_1-cells [25]. Naïve mice that have been target-deleted of the T-bet gene (T-bet KO-mice) spontaneously develop airway remodeling very similar to that seen in asthma and demonstrate multiple functional and inflammatory features characteristic of this disease [26]. Recently, we developed a system consisting of thin lung slices in combination with confocal microscopy, which enables the analysis of ASMC Ca^2+^-signaling in an environment closely resembling the *in vivo *situation [27,28]. In the present study, we applied this system to T-bet KO-mice as a complex and multi-faceted asthma model to study alterations of Ca^2+^-homeostasis in ASMC. As a result, we found that lung slices from T-bet KO-mice preserved the key characteristics of the living animal in terms of increased baseline airway tone (BAT) and BHR. Increased BAT was correlated with an increased incidence of spontaneous changes in [Ca^2+^]_c_, whereas increased BHR correlated with elevated acetylcholine (ACH)-induced Ca^2+^-transients and a higher proportion of ASMC showing Ca^2+^-oscillations. Emptying intracellular Ca^2+^-stores induced higher Ca^2+^-elevations in ASMC from T-bet KO- compared to wild-type mice. We therefore propose that a higher Ca^2+^-content of the intracellular Ca^2+^-stores is involved in the pathophysiology of these changes.

## Methods

Cell culture reagents were obtained from Life Technologies (Eggenstein, Germany). Other reagents were bought from Sigma-Aldrich (Deisenhofen, Germany) unless stated otherwise. Balb/C mice were purchased from Harlan-Winkelmann (Borchen, Germany) and T-bet KO-mice on a Balb/C background from Charles River (Charles River Breeding Labs, Needham, MA). All procedures had been approved by the Ethics Committee of the Ludwig-Maximilians-University Munich.

Lung slices were prepared as described previously [27]. Briefly, after sacrificing the mice, lungs were inflated with 2% agarose-sHBSS and the agarose was gelled by placing the mouse preparation at 4°C. For the use with video-microscopy, slices ~200 μm thick were cut with an EMS-4000 Tissue Slicer (Electron Microscopy Sciences, Fort Washington, PA). For better loading with Ca^2+^-indicator dyes, slices were cut ~100 μm thick for the use with two-photon microscopy. The slices were maintained by floating them in DMEM supplemented with Penstrep^® ^(2.4 ml/l, Penicillin 10.000 U/ml + Streptomycin 10.000 μg/ml) and Fungizone^® ^(4.8 ml/l, Amphotericin B 250 UG/ml) at 37°C in 5 % CO_2_. Experiments were performed on day 2 to 4 of culture and each slice was used for one experiment only. For each group of experiments, slices from 5 to 6 different mice were used.

To measure airway cross-sectional area, lung slices were placed in culture dishes immersed in sHBSS and held in position by a piece of a nylon mesh. Phase-contrast images were recorded using a digital CCD camera (AxioCam MRm, Carl Zeiss Vision, Munich, Germany). Frames were captured in time-lapse (0.5 frame/s) and the cross-sectional area of the airway was measured by pixel summing using the image analysis software "Scion" (Scion Corporation, Frederick, Maryland). The mean cross-sectional area of all airways used was 39326 ± 9390 μm^2 ^without significant differences between T-bet KO- and wild-type mice. This size reflects an airway level below the segmental bronchi and above the respiratory bronchioles. Contraction velocity was defined as maximal change in cross-sectional area per second.

To determine [Ca^2+^]_c _in ASMC, slices were loaded for 1 h at 37°C with 10^-5 ^M Fluo-4-AM (Molecular Probes, Eugene, OR) in sHBSS containing 0.2 % Pluronic (Pluronic F-127, Calbiochem, La Jolla, CA) and 10^-4 ^M sulfobromophthalein. Sulfobromophthalein was used to block unspecific ion pumps and cation channels thereby reducing the loss of de-esterified Fluo-4. After loading, slices were incubated for at least 30 min in sHBSS containing 10^-4 ^M sulfobromophthalein to allow for complete dye de-esterification. The bath solution for all experiments was sHBSS without sulfobromophthalein. The slices were placed in a custom made Plexiglas chamber and held in position by a platinum mesh. Detection of fluorescent signals of single ASMC within the slices was performed with a custom-built two-photon microscope based on an Olympus microscope (BX51WI, Olympus, Hamburg, Germany, for details see [29,30]). Briefly, the 790 nm laser lane of a Ti:Saphir femtosecond laser (Spectra Physics, Darmstadt, Germany) is scanned across the specimen with 2 oscillating mirrors (for X- and Y-scan). The density of photons is only in the focal plane high enough to ensure simultaneous excitation of fluorescent molecules by two photons. Out of the focal plane, the energy of one photon is not sufficient to excite fluorescent molecules and therefore, all emitted light originates from the focal plane. The resultant fluorescence (> 510 nm) is detected by a photomultiplier tube and an image is formed using the recording software "Video Savant" (Cosyco, Germering, Germany). Images were recorded in time lapse-mode (1 f/sec). In the case of ACH-induced Ca^2+^-plateaus, the acquisition rate was increased to 10 f/sec (sufficient to detect Ca^2+^-oscillations with a frequency of up to 500/min) to ensure the detection of potentially high frequency Ca^2+^-oscillations. Regions of interest of 10 × 10 pixels were defined in single ASMC excluding the nucleus and fluorescence intensities were analyzed frame by frame using custom written macros in the image analysis software "Scion". Final fluorescence values were expressed as fluorescence ratio (F/F_o_) normalized to the fluorescence immediately prior to the addition of an agonist (F_o_).

To measure the Ca^2+^-content of the intracellular Ca^2+^-stores, ASMC in lung slices were exposed to 10^-3 ^M caffeine (to open ryanodine-receptor Ca^2+^-channels) or to 10^-6 ^M cyclopiazonic acid (CPA, to inhibit SERCA-pumps), and the resulting increase in cytoplasmic Ca^2+^-concentration was measured. To prevent Ca^2+^-entry by store operated channels, the slices were placed in phosphate-buffered saline without calcium containing 0.02 % EDTA immediately prior to the experiments.

## Statistics

One-way or two-way ANOVA or ANOVA on ranks (combined with pairwise multiple comparisons) were performed using the "Sigma Stat" software (Jandel Scientific, Chicago, IL). A *P *value of less than 0.05 was considered statistically significant.

## Results

### Airway contraction

**Figure 1:**
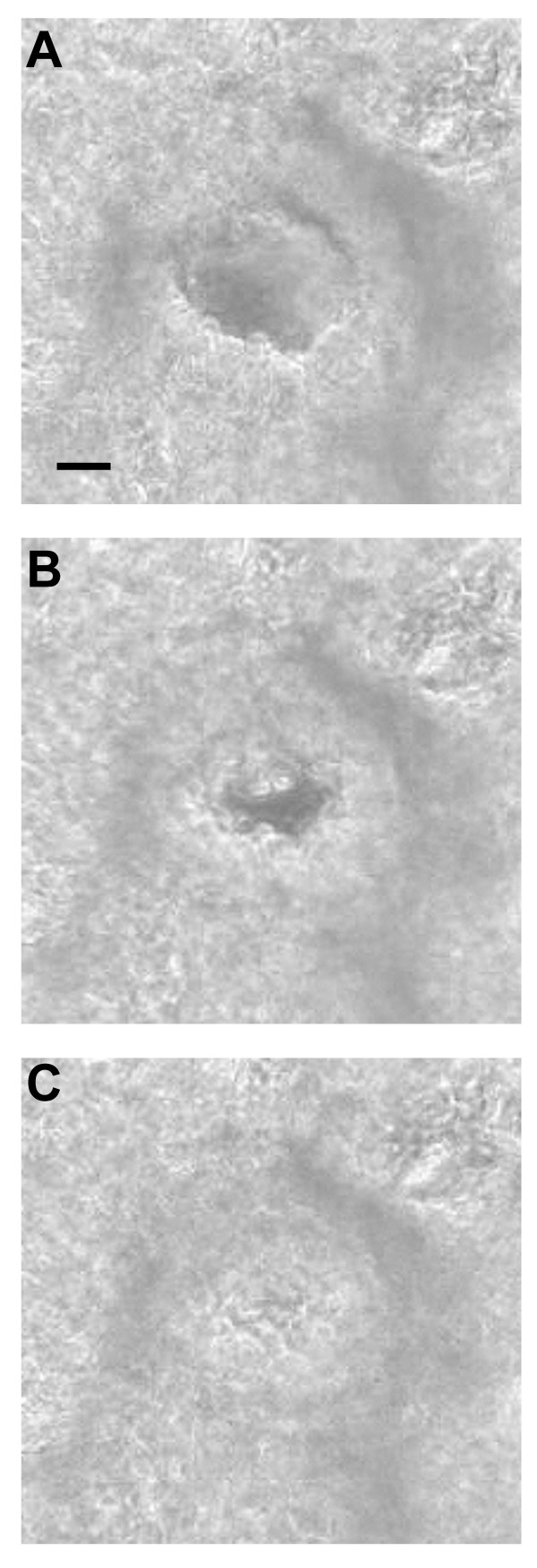
*Appearance of acetylcholine-induced airway contraction in lung slices*. The phase-contrast micrographs show an airway in a lung slice from a T-bet KO-mouse **(A) **immediately before, **(B) **10 sec and **(C) **90 sec after the addition of 10^-7 ^M ACH. Bar: 30 μm [see additional file 1].

**Figure 2:**
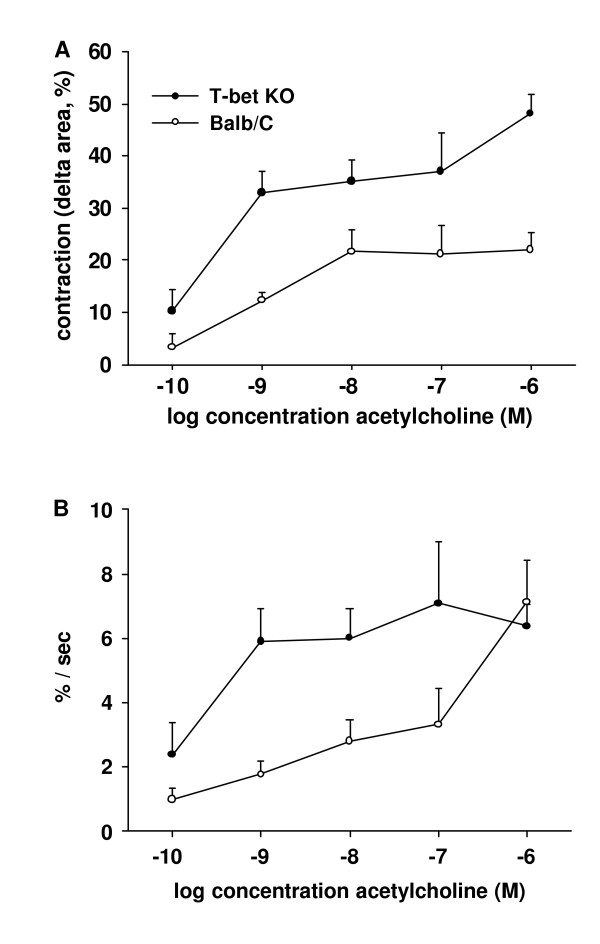
*Bronchial reactivity in lung slices from T-bet KO- and wild-type mice*. Lung slices were exposed to increasing concentrations of ACH and the decrease in cross-sectional area was quantified using digital video-microscopy. **(A) **At all concentrations, the maximal contraction was found to be higher in T-bet KO- (closed circles) compared to wild-type mice (Balb/C, open circles, *P *< 0.001). **(B) **The contraction velocity was also increased in airways from T-bet KO-mice at concentrations from 10^-10 ^to 10^-7 ^M ACH (*P *< 0.01). At 10^-6 ^ACH, contraction velocity (~7 %/sec) was no longer different between the two groups (n = 20 to 25 slices from 5 to 6 mice per concentration).

The addition of ACH led to airway narrowing. In some airways from T-bet KO-mice, even complete airway closure was observed (Fig. 1) [see Additional file 1]. At all concentrations (10^-10 ^to 10 ^-6 ^M), ACH-induced airway contraction was higher in lung slices from T-bet KO-mice compared to wild-type mice (n = 20 to 25 lung slices from 5 to 6 mice per concentration, *P *< 0.001, Figure 2A). The contraction velocity was also higher in airways from T-bet KO-mice at concentrations from 10^-10 ^to 10^-7 ^M ACH (*P *< 0.01, Fig. 2B). At 10^-6 ^ACH, contraction velocity (~7 %/sec) was no longer different between the two groups.

**Figure 3:**
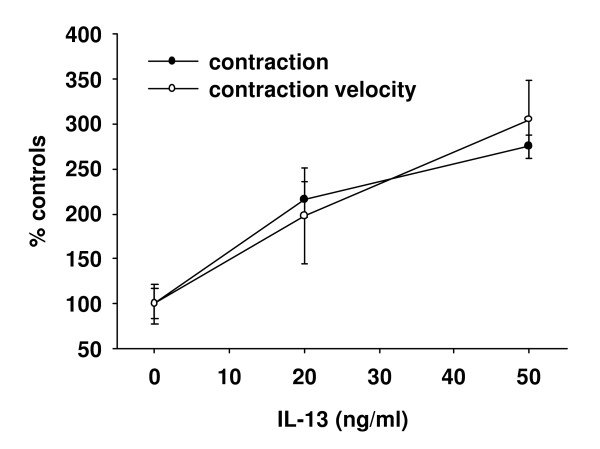
*Effects of IL-13 on bronchial reactivity*. To test if exogenous IL-13 induces BHR, lung slices from Balb/C wild-type mice were exposed to IL-13 and the response to 10^-6 ^M ACH was quantified. Following incubation with IL-13 for 24 h, Ach-induced contraction and contraction velocity increased concentration dependently (n = 15 to 31 slices from 5 to 6 different mice for each data point, *P *< 0.05).

Recently, Finotto et al. reported that the asthmatic changes in T-bet KO-mice are mediated by IL-13 [31]. To test if exogenous IL-13 induces BHR in lung slices, lung slices from Balb/C wild-type mice were exposed to IL-13 and the response to 10^-6 ^M ACH was quantified. Following incubation with 50 ng/ml IL-13 for 24 h, Ach-induced contraction increased to 275 ± 13 % and contraction velocity to 305 ± 44 % of controls (n = 11, *P *< 0.05, Fig. 3).

**Figure 4:**
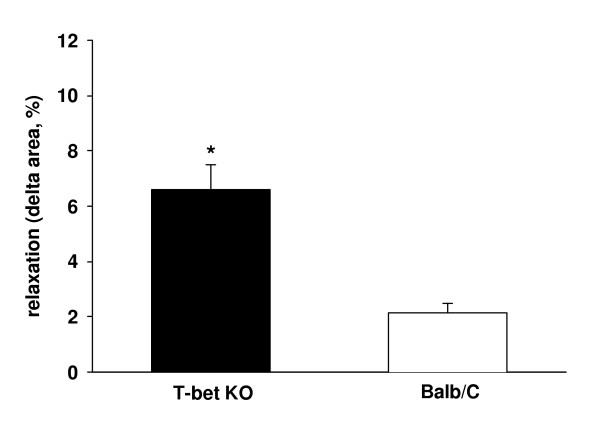
*BAT in lung slices from T-bet KO-mice*. Baseline airway tone was assessed by β-escin induced relaxation of airways in lung slices. Airways in slices from T-bet KO-mice (black columns) showed an increased baseline airway tone compared to airways in slices from wild-type mice (Balb/C, white columns, n = 28 slices from 5 different mice, * = *P *< 0.01).

To measure baseline airway tone (BAT) we used a relaxation solution consisting of 10^-3 ^M β-escin and 10^-7 ^M ATP in phosphate-buffered saline without calcium containing 0.02 % EDTA to permeabilize the cell membrane in zero external calcium. Thereby, the intracellular Ca^2+ ^was removed leading to ASMC relaxation independently from pharmacological properties like e.g. β-adrenergic receptor expression. Without prior contact to contractile agonists, lung slices were exposed to the β-escin relaxation solution and the increase in cross-sectional area was defined as BAT. BAT was found to be higher in airways from T-bet KO-mice compared to wild-type mice (6.6 ± 0.9 % of starting cross-sectional area in T-bet KO-mice *vs *2.1 ± 0.4 % in wild-type mice, n = 28, *P *< 0.01, Fig. 4).

When analyzing the histological appearance of airways in lung sections after H&E staining, an increased thickness of the ASMC-layer in bronchial walls from T-bet KO-mice compared to wild-type mice and infiltration by inflammatory cells could be observed (data not shown, see also [26]).

### Ca^2+^-signaling in ASMC

**Figure 5:**
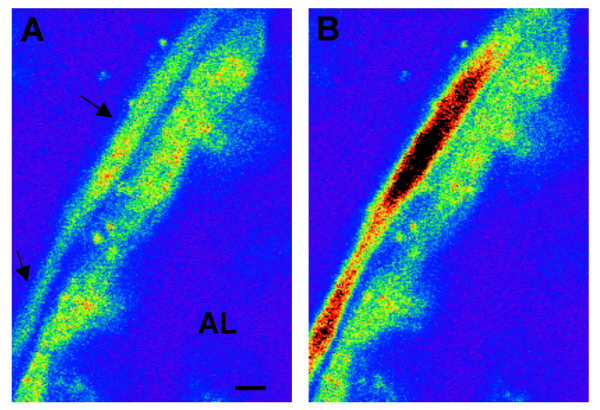
*Appearance of the ACH-induced increase in [Ca*^2+^*]*_c_. The pseudocolor 2-photon micrographs show a part of a bronchial wall of an airway in a lung slice. Airway smooth muscle cells (arrows) are separated from the airway lumen (AL) by epithelial cells. **(A) **shows the airway immediately before and **(B) **5 sec after the addition of 10^-7 ^M ACH. "Warmer" colors indicate higher [Ca^2+^]_c_. Bar = 10 μm [see additional file 2].

**Figure 6:**
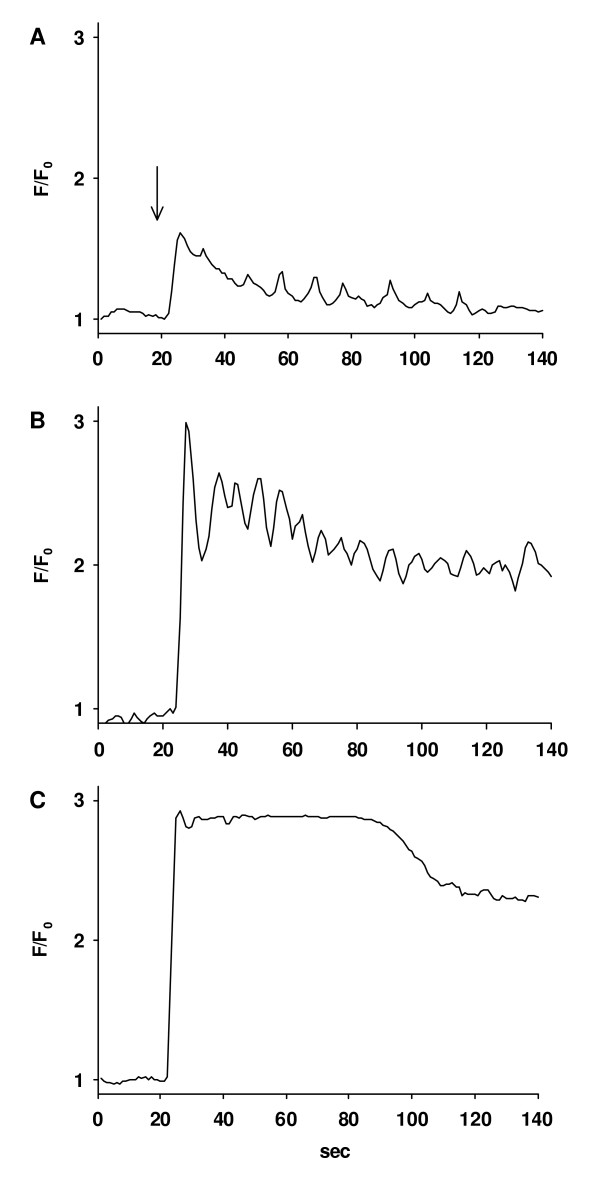
*Representative traces of ACH-induced Ca*^2+^*-signaling*. Regions of interest were defined in ASMC in lung slices and the Ca^2+^-changes in response to ACH (arrow) were expressed as fluorescence ratio F/F_0_. **(A) **At low concentrations (10^-10 ^to 10^-9 ^M, 10^-9 ^M for the trace shown), the Ca^2+^-response consisted of an initial Ca^2+^-transient followed by baseline Ca^2+^-oscillations. **(B) **At higher concentration (≥ 10^-8 ^M, 10^-7 ^M for the trace shown), the Ca^2+^-response consisted of an initial Ca^2+^-transient followed by Ca^2+^-oscillations on an elevated level or **(C) **followed by a Ca^2+^-plateau without Ca^2+^-oscillations (10^-6 ^M for the trace shown).

Ca^2+^-signaling of single ASMC within lung slices was quantified by two-photon microscopy. After addition of ACH, a marked increase in [Ca^2+^]_c _occurred (Fig. 5) [see additional file 2]. This increase consisted of an initial Ca^2+^-transient, which was followed by Ca^2+^-oscillations close to the baseline level in the case of low ACH concentrations (10^-10 ^to 10^-9 ^M, Fig. 6A). At higher concentrations (>= 10^-8 ^M ACH), the initial Ca^2+^-transient was followed by Ca^2+^-oscillations above the baseline level (Fig. 6B) or by a Ca^2+^-plateau without Ca^2+^-oscillations (Fig. 6C).

**Figure 7:**
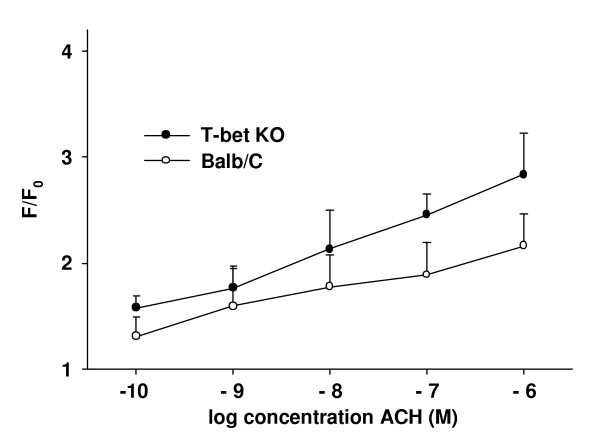
*Magnitude of the ACH-induced Ca*^2+^*-transient*. For all concentrations, the magnitude of the initial Ca^2+^-transient in response to ACH was higher in ASMC from T-bet KO-mice (closed circles) compared to ASMC from wild-type mice (open circles, n = 20 to 26 slices from 5 to 6 different mice per concentration, *P *< 0.05).

**Figure 8:**
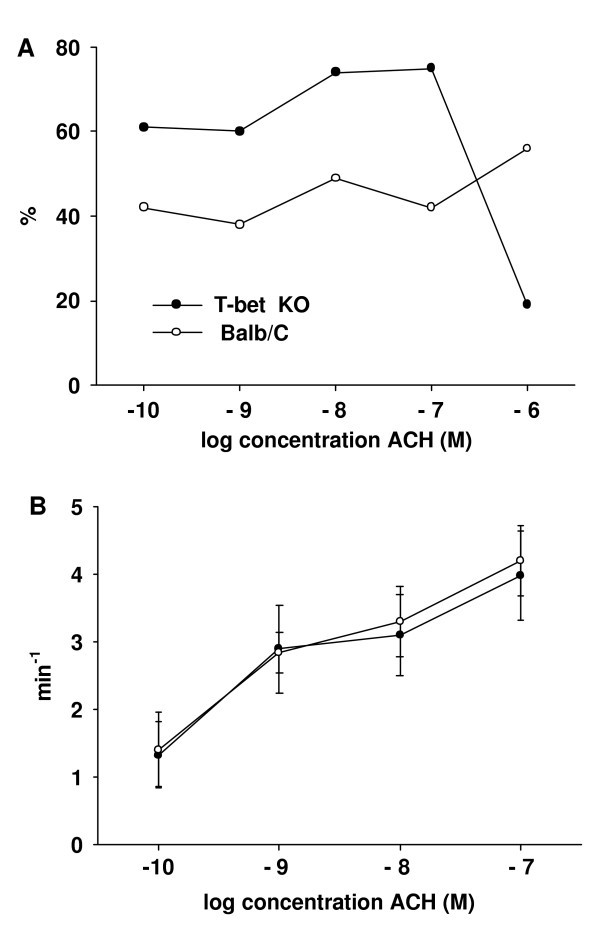
*ACH-induced Ca*^2+^*-oscillations*. **(A) **At ACH-concentrations of 10^-10 ^to 10^-7 ^M, the percentage of ASMC showing Ca^2+^-oscillations in lung slices from T-bet KO-mice (closed circles) was higher compared to wild-type mice (open circles, n = 20 to 26 lung slices from 5 to 6 different mice per concentration, *P *< 0.01). However, at 10^-6 ^M ACH, only 19 % of the ASMC from T-bet KO-mice displayed Ca^2+^-oscillations while 81 % showed a Ca^2+^-plateau following the initial Ca^2+^-transient. **(B) **The frequency of the ACH-induced Ca^2+^-oscillations increased in a concentration dependent manner. No differences between ASMC from T-bet KO-mice and wild-type mice could be observed. Because of the low number of ASMC from T-bet KO-mice displaying Ca^2+^-oscillations at 10^-6 ^M ACH, no frequency was calculated for this concentration.

At all concentrations of ACH, T-bet KO-mice showed a higher magnitude of the initial Ca^2+^-transient compared to wild-type mice (*P *< 0.05, Fig. 7). In addition, the percentage of ASMC displaying Ca^2+^-oscillations differed between the two groups; it was higher in T-bet KO-mice at ACH-concentrations from 10^-10 ^to 10^-7 ^M (*P *< 0.01, Fig. 8A). At 10^-6 ^M, this percentage dropped from 75 % at 10^-7 ^M to 19 % at 10^-6 ^M ACH in T-bet KO-mice, and instead of Ca^2+^-oscillations, 81 % of the ASMC showed a Ca^2+^-plateau after the initial Ca^2+^-transient. Consequently, at 10^-6 ^M ACH the percentage of ASMC displaying Ca^2+^-oscillations was higher in the wild-type mice (56 %). When analyzing the frequency of the Ca^2+^-oscillations, there was a concentration-dependent increase with increasing concentration of ACH in the absence of significant differences between T-bet KO- and wild-type mice (Fig. 8B).

**Figure 9:**
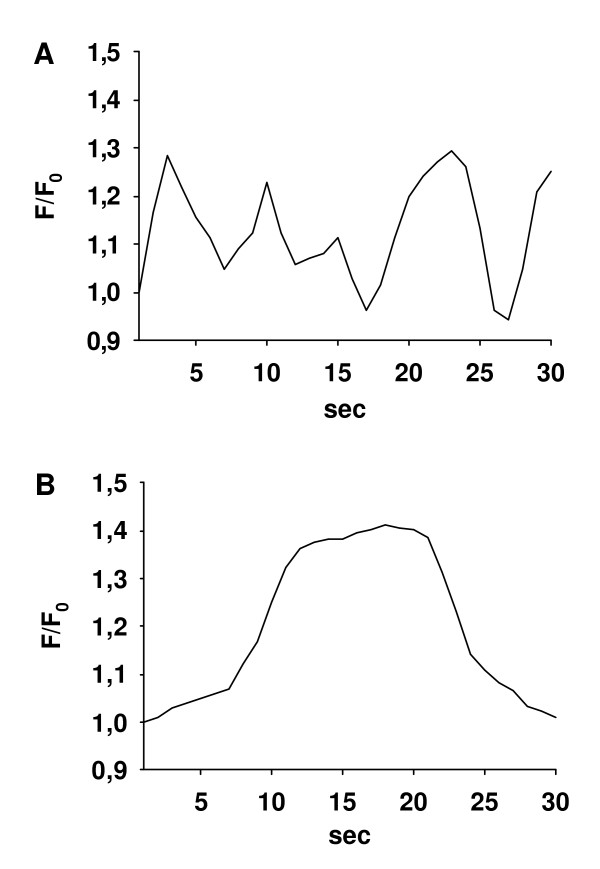
*Representative traces of spontaneous changes in [Ca*^2+^*]*_c_. ASMC in lung slices were recorded and spontaneous changes in [Ca^2+^]_c _were expressed as fluorescence ratio F/F_0_. **(A) **Trace of spontaneous Ca^2+^-oscillations in an ASMC in a lung slice from a T-bet KO-mouse. **(B) **Trace of a spontaneous Ca^2+^-transient in an ASMC in a lung slice from a T-bet KO-mouse.

**Figure 10:**
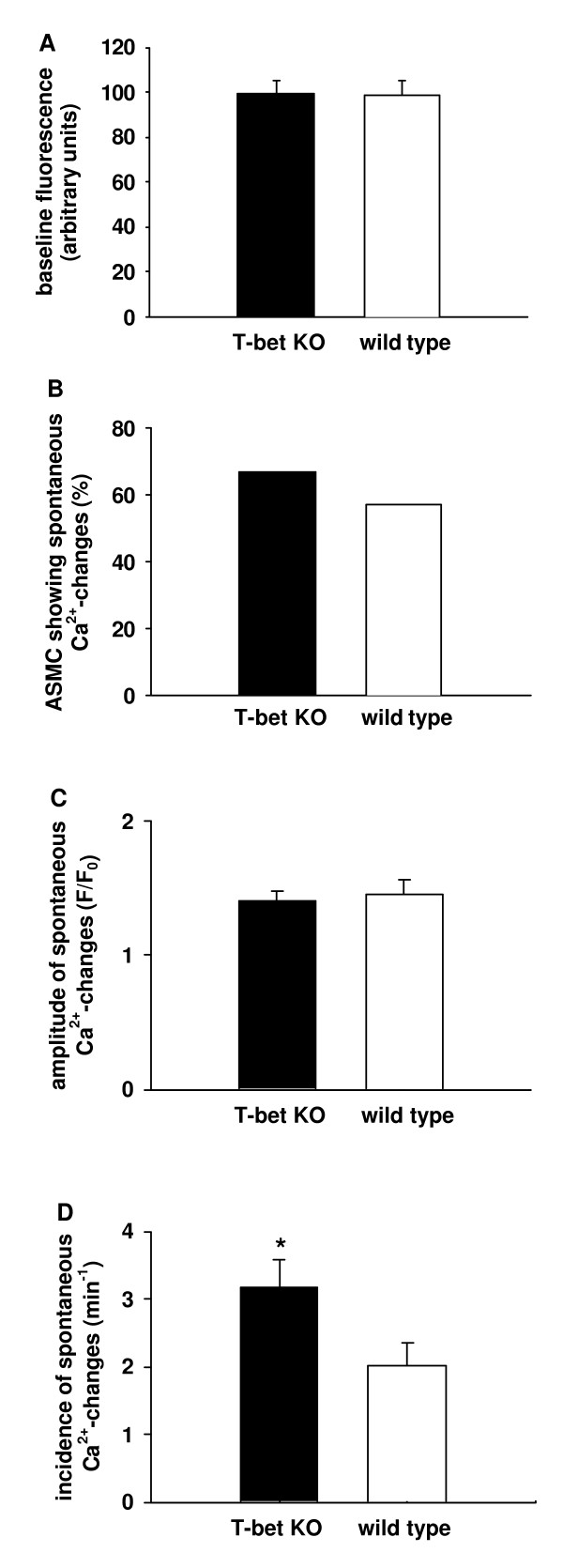
*Spontaneous changes in [Ca*^2+^*]*_c _*in ASMC in lung slices*. Regions of interest were defined in ASMC and spontaneous Ca^2+^-changes recorded using two-photon microscopy. Baseline fluorescence values did not differ between ASMC in lung slices from T-bet KO-mice (black columns) and wild-type mice (white columns). **(B) **The percentage of ASMC displaying spontaneous Ca^2+^-changes also showed no significant differences. **(C) **Furthermore, the amplitude of spontaneous Ca^2+^-changes (expressed as fluorescence ratio F/F_0_) was similar. **(D) **In contrast, the incidence per min of spontaneous Ca^2+^-changes was found to be higher in ASMC from T-bet KO-mice compared to wild-type mice (n = 66 lung slices from 6 different mice for each data point, * = *P *< 0.05).

Without prior contact with contractile agonists, about 60 % of the ASMC showed spontaneous changes in [Ca^2+^]_c_, which occurred as spontaneous Ca^2+^-oscillations or as Ca^2+^-transients (Fig. 9). Baseline fluorescence values did not differ between ASMC in lung slices from T-bet KO- and wild-type mice (Fig. 10A). The percentage of ASMC showing spontaneous changes in [Ca^2+^]_c _was not significantly different between T-bet KO- and wild-type mice (Fig. 10B) nor was the amplitude of these changes (Fig. 10C). However, in those ASMC, which showed spontaneous changes in [Ca^2+^]_c_, the incidence per unit of time of these changes was higher in ASMC from T-bet KO- compared to wild-type mice (3.3 ± 0.4 min^-1 ^in T-bet KO-mice *vs *2.1 ± 0.3 min^-1 ^in wild-type mice; n = 66, *P *< 0.05, Fig. 10D).

**Figure 11:**
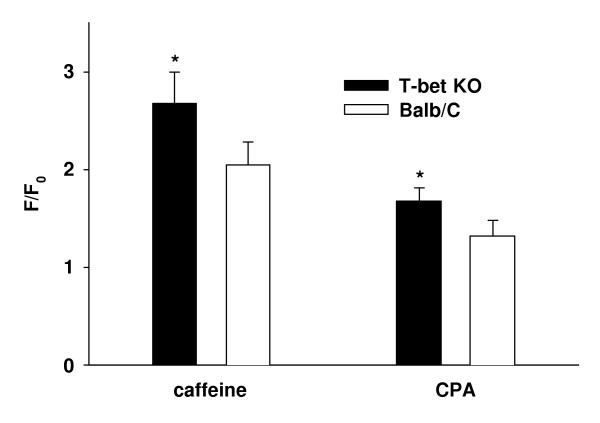
*Ca*^2+^*-content of intracellular Ca*^2+^*-pools*. Ca^2+^-pools of ASMC in lung slices were depleted in the absence of external calcium by either opening ryanodine-receptor Ca^2+^-channels using caffeine or by inhibiting SERCA-pumps using CPA. In both cases, the Ca^2+^-increase following depletion of the Ca^2+^-pools was more pronounced in ASMC from T-bet KO-mice compared to wild -type mice indicating higher Ca^2+^-content in the asthma model (n = 20 lung slices from 5 different mice, * = *P *< 0.05).

To reveal whether an altered Ca^2+^-content of the intracellular Ca^2+^-stores was involved in the altered Ca^2+^-signaling in ASMC from T-bet KO-mice, the Ca^2+^-stores of ASMC in lung slices were depleted by opening ryanodine-receptor Ca^2+^-channels using 10^-3 ^M caffeine or by inhibiting SERCA-pumps using 10^-6 ^M CPA. In both cases, the Ca^2+^-increase following depletion of the Ca^2+^-stores was more pronounced in ASMC from T-bet KO-mice compared to wild-type mice (caffeine: 2.7 ± 0.3 F/F_0 _in T-bet KO-mice *vs *2.0 ± 0.2 F/F_0 _in wild-type mice; CPA: 1.7 ± 0.1 F/F_0 _in T-bet KO-mice *vs *1.3 ± 0.1 F/F_0 _in wild-type mice; n = 20, *P *< 0.05, Fig. 11).

## Discussion

In this study, we investigated the Ca^2+^-homeostasis of ASMC in lung slices from T-bet KO-mice. Based on previous knowledge [26], these mice served as a genetically engineered animal model of asthma. Lung slices from T-bet KO-mice preserved the contractile characteristics of the living mice by showing increased BAT and BHR compared to wild-type mice. BHR could be mimicked incubating lung slices from wild-type mice with IL-13 indicating that the increased levels of IL-13 found in T-bet KO-mice might have mediated the changes in bronchial reactivity (see also [31]). Increased BAT was related to an increased incidence of spontaneous changes in [Ca^2+^]_c_, while BHR correlated with elevated ACH-induced Ca^2+^-transients and a higher proportion of ASMC showing Ca^2+^-oscillations. Baseline fluorescence values and the amplitude as well as the frequency of the ACH-induced Ca^2+^-oscillations were not different. The fact that caffeine and CPA induced higher Ca^2+^-elevations in ASMC from T-bet KO-mice suggests that a higher content of the intracellular Ca^2+^-stores in T-bet KO-mice contributed to the alterations in the Ca^2+^-homeostasis.

The major advantage of lung slices is that the in situ organization of the lung tissue and the contractility of the ASMC are maintained for several days. Lung slices in combination with video-microscopy have been used before to study airway responses [32,33]. Using conventional imaging techniques, observation of ASMC on a single cell-level is prohibited by the thickness of the slice. Therefore, we combined the lung slice technique with confocal microscopy [14,27,28], which allows the observation of single cells in a slice by excluding out of focus light [30]. In this study, we refined this technique by using a custom-build two-photon instead of a confocal microscope, which allowed to reduce photo-bleaching while increasing spatial resolution [29]. With this approach, we were able to combine the advantages of a complex tissue culture system preserving many of the alterations observed *in vivo *with the ability to observe single cell properties, which is usually only rewarded by single cell preparations.

Similar to previous observations [26], we found the thickness of the ASMC-layer to be elevated in histological sections from T-bet KO-mice. Although an increased ASMC mass alone could have accounted for elevated airway contraction, the contraction velocity of airways from T-bet KO-mice was also increased and this finding pointed towards intrinsic alterations in the contractile properties of the ASMC. A thickened ASMC-layer probably affected the stiffness of the airway wall and therefore lung compliance. This may have contributed to the different contractile properties of airways in lung slices from T-bet KO- and wild-type mice. But, we believe that this is an advantage rather than a negative aspect of the lung slice preparation making it even closer to the *in vivo *situation.

Finotto et al. reported elevated levels of TH_2_-cytokines especially IL-13 in the lavage of T-bet KO-mice [26] and recently, the same group showed that IL-13 mediated the asthmatic changes seen in these mice [31]. Furthermore, IL-13 has been shown to increase the Ca^2+^-response to a variety of agonists [19,22]. In our study, incubating lung slices of wild-type mice with IL-13, we found increased contraction and contraction velocity in response to ACH closely resembling the changes seen in lung slices from T-bet KO-mice. To our knowledge, a direct link connecting the genetic alteration in the T-bet gene with BHR has not been established. We therefore propose that the shift from TH_1_- to a TH_2_-phenotype mediated by the loss of the T-bet gene function results in a predominance of TH_2_-cytokines especially IL-13, which in turn mediates BHR.

ASMC from T-bet KO- and wild-type mice showed spontaneous changes in [Ca^2+^]_c_. Depending on the frequency, these changes could be regarded as spontaneous Ca^2+^-oscillations or as spontaneous Ca^2+^-transients, although sometimes the differentiation between slow frequency Ca^2+^-oscillations and Ca^2+^-transients was arbitrary. The higher incidence of changes in [Ca^2+^]_c _in T-bet KO-mice may have led to an increased BAT by either a frequency modulated process integrating Ca^2+^-oscillations or simply by increasing overall [Ca^2+^]_c_.

In a previous study, we proposed that the initial Ca^2+^-transient evoked by ACH determines the level of contraction while the subsequent Ca^2+^-oscillations keep the airway in the narrowed state [27] and this idea has been supported by recent data [34,35]. In accordance with this hypothesis, the augmented Ca^2+^-transient in ASMC from T-bet KO-mice led to increased contraction. However, although the contraction was different, the frequencies of the ACH-induced Ca^2+^-oscillations were similar in T-bet KO- and wild-type mice. After the initial Ca^2+^-transient, the level of [Ca^2+^]_c _has to be high enough to prevent dephosphorylation of MLC and thereby maintaining contraction. The latch state describes a situation, where MLC is dephosphorylated but nevertheless remains attached to actin, which results in a much slower relaxation rate [3]. The present data suggest that once ASMC are in the latch state, comparable frequencies of Ca^2+^-oscillations or a sustained, adequately high steady-state Ca^2+^-plateau may be sufficient to maintain different levels of contraction set by different Ca^2+^-transients.

We found the percentage of ASMC showing ACH-induced Ca^2+^-oscillations to be higher in T-bet KO-mice compared to ASMC from wild-type mice. Even though the frequency of the Ca^2+^-oscillations was comparable, an enhanced recruitment of ASMC showing Ca^2+^-oscillations might have contributed to the maintenance of the higher contraction in ASMC from T-bet KO-mice.

Using two different pharmacological interventions, we found the Ca^2+^-content of intracellular Ca^2+^-pools of ASMC to be elevated in lung slices from T-bet KO- compared to wild-type mice. However, the question arises how an increased Ca^2+^-content of intracellular Ca^2+^-pools of ASMC may be related to the altered spontaneous and ACH-induced Ca^2+^-signaling found in T-bet KO-mice?

An increased Ca^2+^-load of the SR sensitizes both RyR and inositol-3-phosphate receptors (IP_3_R), which leads to increased elemental Ca^2+^-events like Ca^2+^-sparks and Ca^2+^-puffs [8]. The elevated rate of elemental Ca^2+^-events might result in an elevated rate of spontaneous changes in whole cell [Ca^2+^]_c _as observed in ASMC from T-bet-KO mice.

RyR and IP_3_R play a critical role in the induction of Ca^2+^-oscillations because these Ca^2+^-channels repetitively allow or inhibit Ca^2+^-release from the Ca^2+^-pools. Shifting the Ca^2+^-sensitivity of RyR and IP_3_R by increasing the Ca^2+^-content of the Ca^2+^-pools could sensitize the Ca^2+^-signaling apparatus to allow a higher proportion of cells to display Ca^2+^-oscillations in response to ACH as seen in ASMC from T-bet KO-mice.

The ACH-induced initial Ca^2+^-transient has been shown to be caused by Ca^2+^-release from Ca^2+^-pools with Ca^2+^-influx across the cell membrane playing a minor role [27]. Therefore, the increased Ca^2+^-transient in ASMC from T-bet KO-mice presumably was caused by the higher Ca^2+^-load of the Ca^2+^-pools. On the other hand, our results do not preclude the possibility that voltage dependent or independent Ca^2+^-influx contributed to the different Ca^2+^-signaling in T-bet KO-mice.

At the highest ACH concentrations used in our study, ASMC from T-bet KO-mice showed a sustained Ca^2+^-plateau rather than Ca^2+^-oscillations after the initial Ca^2+^-transient. A higher Ca^2+^-load of the Ca^2+^-pools results in a higher Ca^2+^-gradient between the Ca^2+^-pools and the cytoplasm. Ca^2+^-pumps force calcium against this gradient out of the cytoplasm into the Ca^2+^-pools. Therefore, a higher gradient would impair the reuptake of calcium into the Ca^2+^-pools. In the case of Ca^2+^-oscillations with high frequencies and hence short intervals between oscillations, the reduced Ca^2+^-reuptake in-between single oscillations could lead to a Ca^2+^-plateau rather than Ca^2+^-oscillations.

The machinery that establishes Ca^2+^-homeostasis in ASMC is complex and, therefore, it seems unlikely that alteration of a single component, e.g. the content of the Ca^2+^-pools, accounted for all the differences in Ca^2+^-signaling observed in T-bet KO-mice. But, as pointed out, it is plausible that the altered content of the Ca^2+^-pools at least contributed to the increased BAT and BHR of T-bet KO-mice.

A detailed analysis of the Ca^2+^-signaling in asthma seems particularly relevant when considering potential implications for clinical studies. As L-type Ca^2+^-channel-blockers such as nifedipine failed to provide benefits in patients with asthma [36], Ca^2+^-signaling as a therapeutic target has been neglected for a long time. Ca^2+^-influx across the cell membrane via L-type Ca^2+^-channels, however, appears to play a minor role in ASMC [27,37] and this easily explains the failure of L-type Ca^2+^-channel-blockers. On the other hand, the key role of Ca^2+^-homeostasis in ASMC is progressively being acknowledged. We believe that the combination of T-bet KO-mice, lung slices and two-photon microscopy as presented in this study constitutes a promising tool to establish a deeper understanding of the mechanisms underlying the altered Ca^2+^-signaling. A deeper understanding of these mechanisms may lead to development of new drugs specifically targeting the mechanisms of altered Ca^2+^-homeostasis relevant in asthma.

## Conclusion

Lung slices from T-bet KO-mice as an asthma model preserve the key characteristics of the living animal in terms of increased BAT and BHR. Increased BAT is correlated with an increased incidence of spontaneous changes in [Ca^2+^]_c_, whereas increased BHR correlates with elevated ACH-induced Ca^2+^-transients and a higher proportion of ASMC showing Ca^2+^-oscillations. We propose that a higher Ca^2+^-content of the intracellular Ca^2+^-stores is involved in the pathophysiology of these changes. Pharmacological interventions targeting altered Ca^2+^-homeostasis may therefore constitute a new therapeutic tool in asthma.

## Competing interests

The author(s) declare that they have no competing interests.

## Authors' contributions

AB laid out the principal conception and design of the study, carried out experiments on Ca^2+^-signaling in lung slices and drafted the manuscript. JK performed studies on airway contraction in lung slices and contributed substantially to the analysis and interpretation of the data. AK also performed in vitro studies and contributed substantially to the analysis and interpretation of the data. FG revised the manuscript and gave valuable intellectual input in the course of the study. RMH participated in the study design and made substantial contributions to the interpretation of the data. All authors read and approved the final manuscript.

## Supplementary Material

Additional File 1 Two videos showing airway contraction and Ca^2+^-signaling in ASMC in lung slices have been added to the manuscript as online supplement. Click here for file

Additional File 2 Two videos showing airway contraction and Ca^2+^-signaling in ASMC in lung slices have been added to the manuscript as online supplement. Click here for file
